# Cardiac surgery in older patients: hospital outcomes during a 15-year period from a complete national series

**DOI:** 10.1093/icvts/ivab320

**Published:** 2021-11-11

**Authors:** James Mark Jones, Mahmoud Loubani, Stuart W Grant, Andrew T Goodwin, Uday Trivedi, Simon Kendall, David P Jenkins

**Affiliations:** 1 Society for Cardiothoracic Surgery in Great Britain and Ireland, London, UK; 2 Royal Victoria Hospital, Belfast Health and Social Care Trust, Belfast, UK; 3 Hull University Teaching Hospitals NHS Trust, Hull, UK; 4 Division of Cardiovascular Sciences, University of Manchester, UK; 5 James Cook University Hospital, Middlesbrough, UK; 6 Brighton and Sussex University Hospitals NHS Trust, Brighton, UK; 7 Royal Papworth Hospital NHS Foundation Trust, Cambridge, UK

**Keywords:** Older, Valve, Coronary artery bypass grafting

## Abstract

**OBJECTIVES:**

The objective was to review national trends in activity and hospital outcomes in older patients having cardiac surgery over a 15-year time period.

**METHODS:**

Data were collected prospectively and uploaded to the National Institute for Cardiovascular Outcomes Research electronically. Data were validated, cleaned and processed using reproducible algorithms. Mortality was death in hospital after index operation.

**RESULTS:**

A total of 227 442 cardiac procedures were recorded in patients aged ≥70 years of which 46 354 were in those aged ≥80 years. Overall patients aged ≥70 years represented 43% of all adult cardiac surgery in the most recent study year. The annual proportion of surgery in patients ≥80 years increased from 4.1% to 10.8% between the first and last study years. There has been a significant linear increase in octogenarian valve [β 67.44, 95% confidence interval (CI) 55.04 to 79.83, *P* < 0.001] and coronary artery bypass graft surgery (β 32.53, 95% CI 6.16 to 58.90, *P* = 0.020) patients. In-hospital mortality reduced significantly for patients aged 70–79 years (β −0.17, 95% CI −0.20 to −0.13, *P* < 0.001) and all patients aged ≥80 (β −0.37, 95% CI −0.45 to −0.30, *P* < 0.001). The median length of hospital stay was 7 days for 70–79 and 9 days for ≥80 group, compared with 7 days for the whole cohort <70 years.

**CONCLUSIONS:**

This study represents the largest complete validated national dataset of cardiac surgery in the entire population of older patients. Octogenarians represent 11% of adult patients having cardiac surgery by the end of the study period, a three-fold increase from the start. In-hospital mortality in patients aged ≥80 years halved during study period to only 4% despite high logistic EuroSCORE of 15%. Cardiac surgery in octogenarians places a higher demand on resources, however, with an increased postoperative length of stay.

**Figure:**
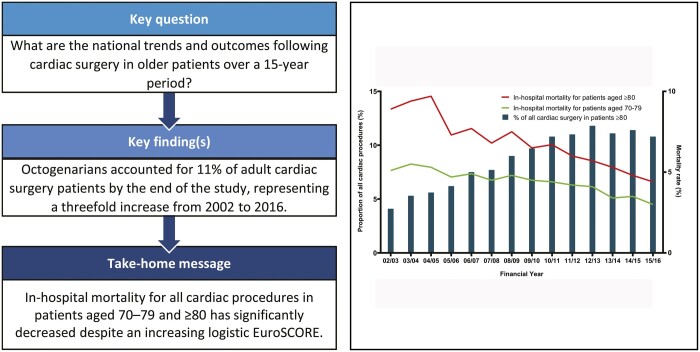

## INTRODUCTION 

The Society for Cardiothoracic Surgery (SCTS) in Great Britain and Ireland first established a cardiac surgery database in 1977 [1]. Following a public inquiry in 2001, these data provided transparency [2]. Risk-adjusted mortality data were placed in the public domain in 2005 in order to reassure all stakeholders that adult cardiac surgery was safe [3]. This database, and associated public reporting strategy, has evolved and in 2011, the National Institute for Cardiovascular Outcomes Research (NICOR) was created.

There have been many developments in treatments for heart disease often at the interface between cardiology and cardiac surgery. The population has aged and patients should not be denied treatment based on age, although after decades of improving life expectancy this trend is slowing [4]. As a result, older patients are being been referred for cardiac surgery, and it is this population where decision-making about risks and benefits of different therapies can be most difficult. The concept of a ‘heart team’ is now embedded in clinical practice and multidisciplinary assessment is undertaken to try to establish the best treatment option for each individual. Treatments for coronary artery disease and aortic stenosis are continuously developing [5–11] and treatment options are increasing in mitral and aortovascular disease. These discussions are of paramount importance in older patients who often have issues of frailty and multiple comorbidities to consider.

It is critical to examine the activity and outcome in older patients in order to inform our practice and recommendations. The objective of this study was therefore to describe trends in activity and in-hospital mortality for patients aged ≥70 undergoing cardiac surgery across a 15-year period in a complete national cohort.

## MATERIALS AND METHODS

### Patients

Reporting of cardiac surgical outcomes has been mandatory in the UK. The NICOR extracted summary data between financial years 2002 and 2016 from the National Adult Cardiac Surgery Audit (NACSA) which included all adult cardiac surgery procedures performed in UK National Health Service hospitals and a small number of hospitals providing private health care as well as hospitals in the Republic of Ireland. A total of 46 centres contributed these data to NICOR. Activity data for all adult cardiac surgery procedures were reviewed to assess the proportion of older patients.

### Ethical statement

As only anonymized summary statistics were utilized for this project, specific ethical approval was not required but the study was approved by both SCTS and NICOR.

### Inclusion and exclusion criteria

Inclusion criteria for this study were any patient aged ≥70 years undergoing cardiac surgery between 2002 and 2016 irrespective of whether the surgery was defined as elective, urgent, emergency or salvage. Exclusion criteria were any procedures not undertaken in cardiac surgery operating theatres or transcatheter aortic valve insertion (TAVI).

### Data processing and analysis

Data were processed, validated and cleaned using reproducible algorithms in accordance with principles published by SCTS previously [12, 13]. Data were handled according to NICOR’s information governance and ethical approvals. Following this process, summaries of the clean data were provided to the research team. All relevant data are within the manuscript.

### Outcomes

The logistic EuroSCORE (predicted in-hospital mortality; European System for Cardiac Operative Risk Evaluation) enabled comparisons in patient risk profiles over time [14]. In-hospital mortality was defined as death following cardiac surgery including patients who died more than 30 days after surgery but without discharge from hospital. Activity and in-hospital mortality were analysed according to financial year for the entire cohort of patients aged ≥70 years. A number of subdivisions were analysed including those aged 70–79 as distinct from those aged ≥80 years. In addition, those undergoing coronary artery bypass grafting (CABG) as distinct from isolated valve surgery were also analysed.

### Statistical analysis

Summary aggregated data for each calendar year were available for analysis. Unadjusted linear regression was used to assess for significant linear trends in summary activity, logistic EuroSCORE and in-hospital mortality over time. The coefficient β and 95% confidence intervals (CIs) were calculated for the linear regression analyses. All statistical analyses were performed using SPSS version 25 (SPSS, Inc., Chicago, IL, USA).

## RESULTS

### Trends in overall activity

**Figure 1:**
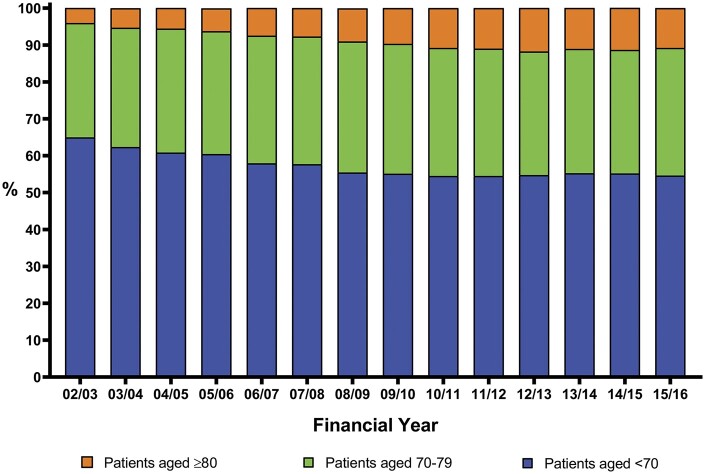
Proportion of all adult cardiac surgery procedures recorded for age groups <70, 70–79 and ≥80 between 2002 and 2016. There has been a significant linear increase in the proportion of all cardiac surgery performed in patients aged ≥80 during the course of the study (*P* < 0.001).

A total of 227 442 cardiac procedures were performed in patients aged ≥70 of which 46 354 were in those aged ≥80. Patients aged ≥70 accounted for 35.0% of the overall adult cardiac surgery workload in 2002 but this rose to 45.4% in 2016. As shown in Table 1, the proportion of adult cardiac surgery performed in patients aged 70–79 has not increased significantly over time (β 0.14, 95% CI −0.22 to 0.29, *P* = 0.085). As shown in Table 2, the proportion of adult cardiac surgery performed in patients aged ≥80 has increased significantly over time (β 0.59, 95% CI 0.47 to 0.72, *P* < 0.001). In 2002, patients aged ≥80 accounted for 4.1% of overall adult cardiac surgery but that has increased to consistently over 10.0% in more recent years. The trends in the proportion of all adult cardiac surgery procedures recorded for age groups <70, 70–79 and ≥80 between 2002 and 2016 are shown in Fig. 1.

**Table 1:** Procedures and outcomes for patients undergoing cardiac surgery aged 70–79 between 2002 and 2016 including percentage of all adult cardiac surgery activity across all ages

Study year	Number of procedures	Mean logistic EuroSCORE	In-hospital mortality rate	Percentage of all adult cardiac surgery activity
2002/2003	10 971	7.9	5.1	31.0
2003/2004	12 282	8.5	5.5	32.3
2004/2005	13 141	8.6	5.3	33.6
2005/2006	13 598	8.9	4.7	33.3
2006/2007	13 412	9.2	4.9	34.6
2007/2008	14 160	9.3	4.5	34.6
2008/2009	14 719	9.7	4.8	35.5
2009/2010	13 617	9.6	4.5	35.2
2010/2011	12 701	9.8	4.4	34.7
2011/2012	12 681	9.9	4.2	34.5
2012/2013	12 226	10.4	4.1	33.5
2013/2014	12 564	10.4	3.4	33.7
2014/2015	12 500	10.7	3.5	33.5
2015/2016	12 516	10.6	3.0	34.6

**Table 2:** Procedures and outcomes for patients undergoing cardiac surgery aged ≥80 between 2002 and 2016 including percentage of all adult cardiac surgery activity across all ages

Study year	Number of procedures	Mean logistic EuroSCORE	In-hospital mortality rate	Percentage of all adult cardiac surgery activity
2002/2003	1444	14.2	8.9	4.1
2003/2004	2032	13.8	9.4	5.3
2004/2005	2176	14.0	9.7	5.6
2005/2006	2534	14.7	7.3	6.2
2006/2007	2912	14.7	7.7	7.5
2007/2008	3174	14.5	6.8	7.7
2008/2009	3731	14.5	7.5	9.0
2009/2010	3744	14.4	6.5	9.7
2010/2011	3971	14.9	6.7	10.8
2011/2012	4050	15.2	6.0	11.0
2012/2013	4311	15.4	5.7	11.8
2013/2014	4130	16.1	5.3	11.1
2014/2015	4244	16.0	4.8	11.4
2015/2016	3903	15.0	4.4	10.8

### Trends in overall risk and hospital outcomes

**Figure 2:**
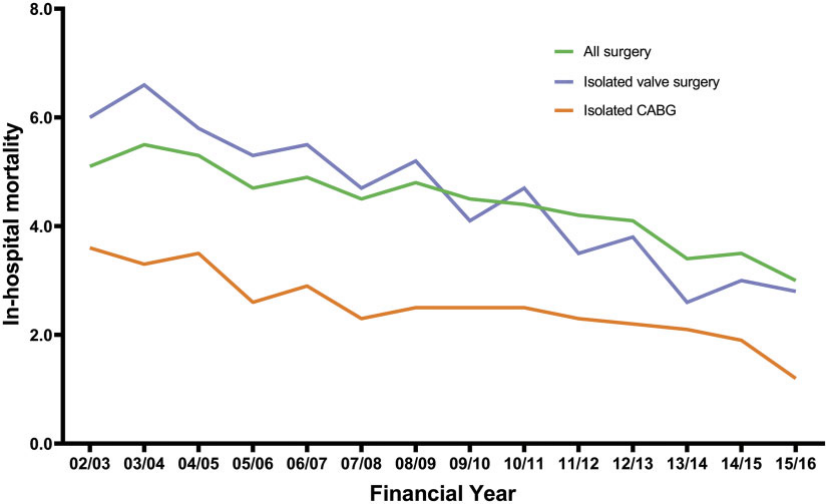
Yearly in-hospital mortality by procedural group for cardiac surgery procedures for patients aged 70–79 between 2002 and 2016. There has been a significant linear decrease in in-hospital mortality for all 3 groups (all *P* < 0.001).

**Figure 3:**
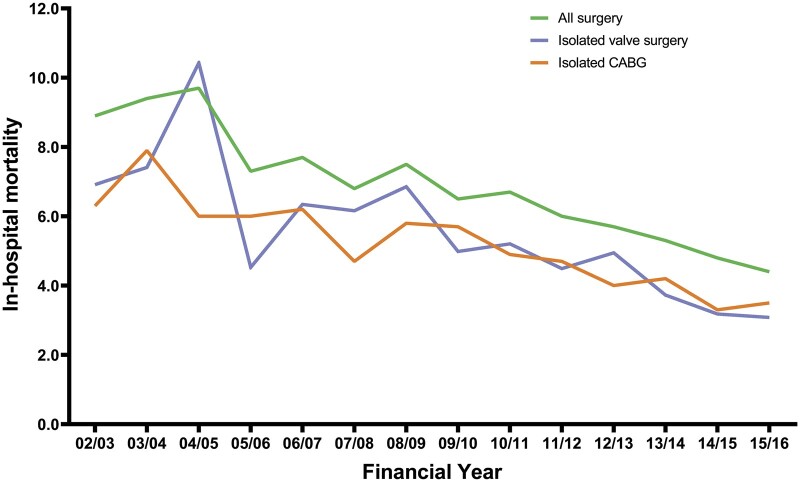
Yearly in-hospital mortality by procedural group for cardiac surgery procedures recorded for patients aged ≥80 between 2002 and 2016. There has been a significant linear decrease in in-hospital mortality for all 3 groups (all *P* < 0.001).

There has been a significant linear increase in mean logistic EuroSCORE for patients aged 70–79 (β 0.20, 95% CI 0.17 to 0.22, *P* < 0.001) and ≥80 (β 0.14, 95% CI 0.08 to 0.19, *P* < 0.001). In patients aged 70–79, the mean logistic EuroSCORE has increased from 7.9 at the start of the study period to 10.6 in the last year of the study, as shown in Table 1. In patients aged ≥80, the mean logistic EuroSCORE was 14.2 in the first year of the study and 15.0 in the last year of the study, as shown in Table 2. Despite the increase in mean logistic EuroSCORE, in-hospital mortality for those aged 70–79 has significantly reduced over the study period (β −0.17, 95% CI −0.20 to −0.13, *P* < 0.001) as shown in Fig. 2. In the first year of the study, in-hospital mortality in this group was 5.1% and in the most recent year, it was 3.0%. As shown in Fig. 3, in-hospital mortality has also significantly fallen in patients aged ≥80 from 8.9% in the first year of the study to 4.4% in the most recent year (β −0.37 95% CI −0.45 to −0.30, *P* < 0.001). The median length of hospital stay was 7 days for 70–79 and 9 days for >80-year group by the end of the study period, compared with 7 days for whole cohort <70 years.

### Isolated coronary artery bypass grafting in older patients

During the study period, 107 601 patients aged ≥70 underwent CABG with 15 353 of these patients aged ≥80. As shown in Table 3, there has been a consistent decrease in the number of patients aged 70–79 undergoing isolated CABG (β −263.53, 95% CI −349.94 to −177.13, *P* < 0.001), although when expressed as a proportion of all adult patients having CABG, this was not significant (β 0.04, 95% CI −0.14 to 0.22, *P* = 0.65). As shown in Table 4, there has been a consistent increase in both the number (β 32.53, 95% CI 6.16 to 58.90, *P* = 0.020) and proportion of patients aged ≥80 undergoing isolated CABG (β 0.39, 95% CI 0.29 to 0.50, *P* < 0.001). Despite a significant linear increase in mean logistic EuroSCORE (β 0.06, 95% CI 0.01 to 0.10, *P* = 0.015), the in-hospital mortality for patients undergoing isolated CABG aged 70–79 has decreased (*P* < 0.001) and was 1.2% for the most recent year of the study. There has also been a significant linear increase in mean logistic EuroSCORE for patients aged ≥80 (*P* = 0.020) with a decrease in in-hospital mortality (*P* < 0.001).

**Table 3:** Isolated CABG procedures and outcomes for patients aged 70–79 between 2002 and 2016 including percentage of all isolated CABG activity across all ages

Study year	Number of procedures	Mean logistic EuroSCORE	In-hospital mortality rate	Percentage of all isolated CABG activity
2002/2003	7055	5.9	3.6	29.9
2003/2004	7661	6.3	3.3	31.5
2004/2005	7940	6.3	3.5	32.3
2005/2006	7971	6.3	2.6	32.8
2006/2007	7603	6.7	2.9	34.2
2007/2008	7843	6.5	2.3	33.5
2008/2009	7557	6.8	2.5	34.1
2009/2010	6850	6.8	2.5	34.0
2010/2011	6243	6.7	2.5	33.5
2011/2012	5528	6.9	2.3	32.7
2012/2013	5139	7.3	2.2	31.7
2013/2014	5006	7.0	2.1	31.9
2014/2015	4942	7.0	1.9	31.7
2015/2016	4910	6.2	1.2	32.6

CABG: coronary artery bypass grafting.

**Table 4:** Isolated CABG procedures and outcomes for patients aged ≥80 between 2002 and 2016 including percentage of all isolated CABG activity across all ages

Study year	Number of procedures	Mean logistic EuroSCORE	In-hospital mortality rate	Percentage of all isolated CABG activity
2002/2003	587	11.0	6.3	2.5
2003/2004	849	11.3	7.9	3.5
2004/2005	856	10.6	6.0	3.5
2005/2006	985	12.0	6.0	4.1
2006/2007	1054	12.1	6.2	4.7
2007/2008	1209	12.2	4.7	5.2
2008/2009	1297	11.0	5.8	5.9
2009/2010	1362	11.6	5.7	6.8
2010/2011	1351	11.9	4.9	7.3
2011/2012	1230	12.4	4.7	7.3
2012/2013	1289	12.5	4.0	8.0
2013/2014	1150	12.8	4.2	7.3
2014/2015	1058	12.4	3.3	6.8
2015/2016	1076	11.6	3.5	7.1

CABG: coronary artery bypass grafting.

### Valve surgery in older patients

During the study period, 51 380 patients aged ≥70 underwent isolated valve surgery with 13 578 of these patients aged ≥80. There has been a consistent increase in the number of patients aged 70–79 undergoing isolated valve surgery ranging from 1948 patients in the first year up to 3261 patients in the most recent year analysed (β 74.13, 95% CI 42.74 to 105.53, *P* < 0.001). When expressed as a proportion of all adult patients having isolated valve surgery, there has also been a significant linear increase in patients aged 70–79 (β 0.41, 95% CI 0.28 to 0.53, *P* < 0.001). As shown in Table 5, there has been a consistent increase in both the number (β 67.44, 95% CI 55.04 to 79.83, *P* < 0.001) and proportion (β 0.68, 95% CI 0.54 to 0.82, *P* < 0.001) of patients aged ≥80 who undergo isolated valve surgery. The mean logistic EuroSCORE for isolated valve surgery has not significantly changed for patients aged 70–79 (β 0.03, 95% CI −0.04 to 0.09, *P* = 0.41) or aged ≥80 (β 0.07, 95% CI −0.05 to 0.19, *P* = 0.20) but the in-hospital mortality has significantly fallen for patients aged 70–79 (β −0.29, 95% CI −0.35 to −0.23, *P* < 0.001) and aged ≥80 (β −0.37, 95% CI −0.55 to −0.19, *P* = 0.001) as shown in Figs  2 and 3. The in-hospital mortality rates for isolated valve surgery in the most recent year of the study for patients aged 70–79 and ≥80 were 2.8% and 3.1%, respectively.

**Table 5:** Isolated valve procedures and outcomes for patients aged ≥80 between 2002 and 2016 including percentage of all isolated valve surgery across all ages

Study year	Number of procedures	Mean logistic EuroSCORE	In-hospital mortality rate	Percentage of all isolated valve surgery
2002/2003	434	14.8	6.9	6.8
2003/2004	553	13.9	7.4	7.7
2004/2005	652	15.4	10.4	8.8
2005/2006	751	14.0	4.5	9.3
2006/2007	804	14.8	6.3	10.2
2007/2008	828	15.1	6.2	9.7
2008/2009	1094	15.9	6.9	11.8
2009/2010	1063	15.0	5.0	11.8
2010/2011	1191	15.4	5.2	14.0
2011/2012	1158	14.7	4.5	14.2
2012/2013	1253	15.8	4.9	15.4
2013/2014	1181	16.8	3.7	14.7
2014/2015	1351	15.9	3.2	15.7
2015/2016	1265	13.8	3.1	13.9

## DISCUSSION

Against the trend of an overall reduction in younger patients, there has been a sustained increase in cardiac surgery in older patients. The proportion of patients undergoing surgery aged ≥70 has increased over time and now makes up ∼45% of all patients undergoing adult cardiac surgery. This increase has largely been driven by an increase in octogenarians undergoing surgery.

As the age of patients has increased over time, comorbidity measured using the logistic EuroSCORE has increased across all older patients and older patients undergoing isolated CABG. The logistic EuroSCORE has remained relatively stable in older patients undergoing isolated valve surgery. Despite the increases in measured risk, the in-hospital mortality rate across all procedures and operative urgencies has fallen to just 3% for patients aged 70–79 by the end of the study period. The in-hospital mortality rate in octogenarians has halved over the course of the study and was only 4.4% in the last year despite a persistently high mean logistic EuroSCORE.

These trends are consistent with previously reported trends in cardiac surgery of increasing age and falling in-hospital mortality associated with the passage of time [15]. This improvement is probably multifactorial and almost certainly includes improvements in perioperative management and anaesthetic care. Given the increase in life expectancy, it is also likely that patients may be fitter for their age at the end of the study period compared to the beginning.

Although age contributes to the calculation of the logistic EuroSCORE, older patients often have additional comorbid factors and may require more extensive operations. The logistic EuroSCORE was developed on retrospective data and designed to predict the risk of in-hospital mortality prospectively. This paper highlights the trend of improving survival after cardiac surgery despite an increasing mean logistic EuroSCORE. Thus, it would appear that the national database, which was developed for quality assurance, is associated with quality improvement. There will be a limit to this quality improvement, but during the period of improvement, historical models such as the logistic EuroSCORE will tend to over predict the risk of mortality. For this reason, in clinical governance analyses of cardiac surgery conducted through NICOR and SCTS, the baseline logistic EuroSCORE has continued to be recalibrated to ensure appropriate quality benchmarks [16].

Older patients often require longer postoperative stays and therefore an increase in resources compared to younger patients [17]. By the end of the study, the median length of stay for patients aged over 80 was 9 days compared to 7 days for those aged under 80. The patterns demonstrated were broadly consistent regardless of whether patients were undergoing CABG or valve surgery.

Interventions such as percutaneous coronary intervention (PCI) undoubtedly affect decision-making as to the optimal revascularization strategy for any individual patient. There was a decrease in the number of patients aged 70–79 undergoing isolated CABG although when expressed as a proportion of all adult patients having CABG, this was not significant. However, the consistent and significant increase in the number of patients aged ≥80 undergoing isolated CABG was significant, when expressed as a proportion of all adult patients having CABG. It is possible that a clinical practice strategy could account for this noted fall in younger patients undergoing CABG, in favour of initial PCI while deferring cardiac surgery until patients are older. Clear guidelines relating to PCI and CABG as revascularization strategies continue to be important [6, 7]. Despite the increase in mean logistic EuroSCORE, the in-hospital mortality for patients of all operative urgencies undergoing isolated CABG aged 70–79 has significantly decreased and was only 1.2% for the most recent year of the study. Similar findings applied to patients aged ≥80.

Less invasive valve treatment options may also result in patients now being referred for cardiac surgery who may not have been considered in the past [10, 11]. There was a consistent increase in the number of patients aged 70–79 undergoing isolated valve surgery with similar findings in patients aged ≥80. It is likely that the impact of these less invasive options will increase in the future. TAVI was reserved initially for a high-risk cohort of patients and many of these were elderly but there is increasing discussion regarding the validity of utilizing TAVI in patients with severe aortic stenosis at intermediate and low risk [8, 9]. The Society of Thoracic Surgeons database records increased numbers of patients undergoing transcatheter aortic valve replacement while there is a reduction in patients having isolated surgical AVR and combined AVR and CABG [5]. The opportunity of patient choice between TAVI or conventional AVR may have influenced the number and age of patients undergoing AVR in the latter part of the period studied in this complete national cohort. Another consideration is the availability of rapid deployment valves, which may affect decision-making around the choice of prosthesis in elderly patients undergoing aortic valve surgery [18, 19]. This may help to account for the observation that the mean logistic EuroSCORE for isolated valve surgery did not significantly change for patients aged 70–79 or aged ≥80. However, the in-hospital mortality fell significantly in both groups. The in-hospital mortality rates for isolated valve surgery of all operative urgencies in the most recent year of the study for patients aged 70–79 and ≥80 were 2.8% and 3.1%, respectively. This would confirm that despite other valuable treatment modalities available to treat abnormal heart valves, surgery is still associated with excellent in-hospital mortality in older patients. This is consistent with a study of 2005 patients aged ≥80 which also documented median survival of 7.1 years following conventional AVR and functional improvement in 90% of survivors [20].

The SWEDEHEART (Swedish Web-system for Enhancement and Development of Evidence-based care in Heart disease Evaluated According to Recommended Therapies) 2016 Annual Report shows that the proportion of patients aged over 70 years is similar to that in the UK and Ireland. However, in the UK and Ireland, the proportion of patients over 80 years of age undergoing cardiac surgery is higher [21].

The confirmation that older patients are being been referred for cardiac surgery is important and of interest to all stakeholders in the field of cardiac surgery. Decision-making concerning risks and benefits of different therapies can be most difficult in this population. The concept of a ‘heart team’ is now established in the management of patients with coronary artery disease and valve pathology [22, 23].

Octogenarians with prolonged intensive care unit length of stay have been reported to have acceptable functional survival at 1 year, although they had high rates of early re-hospitalization [24]. Longer-term survival and quality of life analyses have demonstrated good results 10 years after surgery although improvements in quality of life were less pronounced and survival was lower in patients >75 years [25]. It is not surprising that frailty as assessed by slower gait speed was associated with increased odds of major morbidity or mortality [26].

Multidisciplinary assessment is undertaken to try to establish the best treatment option for each individual. These discussions are of paramount importance in older patients who often have issues of frailty and multiple comorbidities to consider. This study has demonstrated that over a prolonged 15-year period representing the entire national cohort, increasing numbers of older patients have had successful hospital outcomes from cardiac surgery. Despite high comorbidity, the in-hospital mortality has fallen although the demand for resources is higher as reflected in the prolonged hospital stay.

### Strengths and limitations

The evident value of this study which represents the largest complete validated national dataset of cardiac surgery in older patients is that patient inclusion is not subject to selection bias. Despite the obvious strengths to the study, there are a number of limitations. Only aggregated summary data were available for analysis and as the objective of the study was to present and broadly describe data over time more complex trend analyses have not been performed. The indication for surgery and the inclusion of patients are subject to a ‘real world’ situation and due to the nature of the extensive validation data is not available for the most recent years. Moreover, we have not included data on patients who underwent PCI or TAVI. The assessment of complication rates, longer-term survival and quality of life analysis has not been possible in this study.

However, the study documents the outcomes for a complete national cohort of 227 442 patients aged 70 years and over who had cardiac surgery over a 15-year period. We have also reviewed subgroups of those aged 70–79 as well as patients aged ≥80 for cohorts undergoing CABG as well as those undergoing isolated valve surgery. The risk profile of patients as reflected in the EuroSCORE was presented along with the outcomes in terms of in-hospital mortality and hospital stay.

## CONCLUSIONS

This study represents the largest complete validated national dataset of cardiac surgery in the population of older patients. The mean age of patients undergoing all cardiac surgery has increased with time. Octogenarians represented 11% of patients undergoing adult cardiac surgery by the end of the 15-year study period, almost a three-fold increase from the start of it.

In-hospital mortality in patients aged over 80 years halved during study period to only 4% despite high comorbidity with a logistic EuroSCORE of 15%. Excellent outcomes were achieved at the cost of a higher demand on resources as evidenced by a median hospital stay of 9 days. Although this report is limited to survival in hospital, it will also be important to evaluate the impact on longer-term survival and quality of life in this age group.

## Notes

Presented at the 34th Annual Meeting of the European Association for Cardio-Thoracic Surgery, Barcelona, Spain, 8–10 October 2020.

## ABBREVIATIONS

AVR: Aortic valve replacement
CABG: Coronary artery bypass grafting
CI: Confidence interval
NICOR: National Institute for Cardiovascular Outcomes Research
PCI: Percutaneous coronary intervention
SCTS: Society for Cardiothoracic Surgery
TAVI: Transcatheter aortic valve insertion
